# Downregulation of CFTR Is Involved in the Formation of Hypertrophic Scars

**DOI:** 10.1155/2020/9526289

**Published:** 2020-01-02

**Authors:** Yiwen Zhou, Yixuan Zhao, Hengyu Du, Yinjun Suo, Hao Chen, Haizhou Li, Xiao Liang, Qingfeng Li, Xiaolu Huang

**Affiliations:** ^1^Department of Plastic & Reconstructive Surgery, Shanghai Ninth People's Hospital, Shanghai Jiao Tong University School of Medicine, 639 Zhizaoju Road, Shanghai 200011, China; ^2^Institute of Biomedical and Pharmaceutical Sciences, Guangdong University of Technology, Guangzhou Higher Education Mega Center, Panyu District, Guangzhou, China

## Abstract

Hypertrophic Scars (HTSs) are a complex fibroproliferative disorder, and their exact mechanism is still not fully understood. In this study, we first found that cystic fibrosis transmembrane conductance regulator (CFTR) expression was downregulated in human hypertrophic scars at the RNA and protein levels by microarray data analysis, RT-PCR, and immunofluorescence (IF) staining. To validate that this downregulation of CFTR is involved in the formation of HTSs, we then applied a mechanical overloading intervention in both wild type and CFTR-mutant mice (ΔF508). Our results showed thatΔF508 mice exhibited delayed wound healing and a significantly larger HTS on day 28. Masson staining revealed that there was more collagen deposition in the HTS, and Sirius red staining and IF staining showed a higher ratio of collagen 1/collagen 3 (Col1/Col3) in ΔF508 mice. Real-time RT-PCR showed that the proinflammatory markers were higher in ΔF508 mice in all phases of scar formation, whereas the proliferation marker was similar. Moreover, we harvested the fibroblasts from both mice. Western blotting showed that the expression of Col1 was the same in both mice, and the expression of Col3 was significantly lower in ΔF508 mice. However, in a mechanical overloading condition, the expression of Col1 was significantly higher in ΔF508 mice, and the expression of Col3 was the same in both mice. Taken together, our results indicate that the downregulation of CFTR might affect the function of fibroblasts, resulting in a lower level of collagen type 3 and a higher ratio of Col1/Col3, and thus aggravate the formation of HTSs in mechanical overloading conditions.

## 1. Introduction

Hypertrophic scarring is a type of dermal fibroproliferative condition resulting from a pathological wound healing process after burns, severe trauma, or surgical procedures [1]. Usually, hypertrophic scars (HTSs) are red, inflamed, itchy, raised, rigid, and even painful [2]. Histologically, they are characterized by excessive deposition of collagen in the dermis, which results from an imbalanced production and degradation of the extracellular matrix (ECM) [3].

Among various scar types, HTSs have an incidence between 4.5% and 16% in the general population [4], and approximately 35% of surgical skin wounds result in HTSs after 1 year [5, 6]. Although HTSs are not life threatening, they can cause several functional and cosmetic problems, resulting in a serious burden for patients [7].

Abundant studies on hypertrophic scars have been conducted in recent years, while the mechanism underlying the formation of HTSs remains complex and is not fully understood. Specifically, several factors have been demonstrated to play a dominant role in human HTS formation, including mechanical overload [8], local inflammation [9], and fibroblast activation [10].

Cystic fibrosis transmembrane conductance regulator (CFTR), a cAMP-dependent anion channel, is a crucial pathogenic gene related to cystic fibrosis (CF). It was recently recognized that in addition to transporting anions, CFTR is involved in other biological processes, including inflammation [11], cell proliferation [12], cell differentiation [13], and wound healing [14, 15]. In particular, several studies have demonstrated that CFTR is expressed in mouse skin and is first diminished and then reappears during wound healing. Moreover, CFTR deficiency could cause delayed skin wound healing [14, 15]. As HTSs result from abnormal wounding healing, we hypothesized that the downregulation of CFTR might also be involved in the formation of hypertrophic scars.

In the current study, we aimed to examine whether the expression of CFTR is downregulated in human HTS tissues and to demonstrate whether it is involved in the formation of HTSs using CFTR-mutation mice and a mechanical overloading-induced HTS model.

## 2. Materials and Methods

### 2.1. Tissue Collection and Microarray Data

Samples of normal human skin and hypertrophic scars were obtained from Shanghai Ninth People's Hospital with ethics approval from the Human Research Ethics Committee of Shanghai Jiao Tong University School of Medicine in accordance with the Declaration of Helsinki. Written informed consent for sample collection was obtained from patients undergoing surgery. Microarray data were generated from hypertrophic scar tissues (*n* = 9) and normal skin tissues (*n* = 3), and data for CFTR was extracted.

### 2.2. Real-Time RT-PCR

According to the manufacturer's instructions, TRIzol reagent (Invitrogen; Thermo Fisher Scientific, Inc., Waltham, MA, USA) was used to isolate total RNA from tissues and cells. RNA concentration and purity were measured at 260 nm absorbance and the ratio of absorbance at 260/280 nm was recorded on a NanoDrop2000 spectrophotometer (Thermo Fisher Scientific, Inc.). In all RNA samples, the synthesized cDNA was analyzed with RT-qPCR using SYBR® Premix (Takara Biotechnology Co., Ltd., Dalian, China) and the 7900 HT fast real-time q-PCR system (Thermo Fisher Scientific, Inc.). RT-qPCR cycling conditions were as follows: 40 cycles each including 5 sec of denaturation at 95°C and 30 sec of annealing/extension at 60°C. GAPDH was utilized as a reference gene. The gene expression data were analyzed using the 2^−ΔΔCT^ method (25). The primer sequences used are listed in Table 1. Each experiment was replicated three times.

### 2.3. Animals

Male CFTR-mutation (ΔF508) mice have a mutant CFTR gene with a deletion of phenylalanine at residue 508, which corresponds to the most frequent CFTR-mutation in CF patients. C57BL/6 and ΔF508 mice were purchased from the Laboratory Animal Service Center of the Chinese University of Hong Kong. The Guide for the Care and Use of Laboratory Animals and the ARRIVE guidelines were followed in all animal procedures. The Animal Ethics Committee of the Chinese University of Hong Kong approved the study protocol, and all animal experiments were conducted in accordance with the university guidelines. Animals were kept in a temperature-controlled room with a 12 h light/12 h dark cycle and had free access to food and water.

### 2.4. Mechanical Overloading-Induced HTS Model

Based on our previous studies, the model of HTS was built by a mechanical overloading method according to the protocol provided by Arabi et al. [8]. Briefly, the mice were anaesthetized by intraperitoneal injection of ketamine (75 mg/kg) and xylazine (10 mg/kg), and 2.5 × 10 mm full-thickness wounds were made on the dorsal midline. A silicon splint (5 × 20 mm internal, 15 × 30 mm external), with instant-bonding adhesive spread on its edges, was placed around the wound and secured to the skin by interrupted sutures. To apply mechanical tensions that induced hypertrophic scars, an expansion screw was also fixed on the edges of the splint by sutures. Subsequently, wounds were dressed with a self-adhering elastic bandage. Mice were placed on a warm pad until they were woken up and treated with analgesics (buprenorphine, 0.05 mg/kg, every 12 h for 7 days). Mice were housed in individual cages in a clean facility and checked daily to ensure that the bandage remained in place. Four days after surgery, mechanical tension across the wound was inflicted by the expansion screw. On days 0, 7, 14, 21, and 28, photos of the wound and scar areas were captured. Mice were sacrificed at sample collection.

### 2.5. Cell Culture

Full-thickness skin on the dorsal side was harvested from 8-week-old wild type or ΔF508 mice. Skin tissue was digested in Dispase II (2 mg/ml, cat No. 4942078001, Roche, USA) for 6 h at room temperature to separate the dermis and epidermis. Then, the dermal tissue was digested in Collagenase I (2 mg/ml, cat No. C7657, Sigma, USA) for 1 h at 37°C and strained through a 40 *μ*m cell mesh. Primary fibroblasts were seeded at a density of 1 × 10^5^/ml and were cultured in Dulbecco's Modified Eagle Medium (DMEM; Gibco, NY, USA) supplemented with 10% fetal bovine serum (FBS; Gibco, NY, USA) and 1% Penicillin Streptomycin (PS; Gibco, NY, USA). Fibroblasts were cultured in a 37°C incubator with 5% CO_2_. The culture medium was changed every other day.

### 2.6. Cell Stretching

Fibroblasts were plated in flexible silicone chambers coated with type-I collagen at 1 × 10^5^ cells/mL. Cyclic mechanical tension was applied for 24 hours (1 Hz, 10% elongation) using a cell stretching system (STB-140, STREX Osaka, Japan). Cells were harvested immediately after mechanical stretching was complete. Control cells were cultured in the same chambers and incubators without the application of tension.

### 2.7. Western Blot Analysis

Total protein lysates of skin samples and cells were prepared with radioimmunoprecipitation assay (RIPA) buffer containing a protease inhibitor cocktail (Roche, Basel, Switzerland). Equal amounts of the protein from each sample were loaded into an 8% SDS polyacrylamide gel and transferred to 0.45 *μ*m polyvinylidene difluoride (PVDF) membranes. The PVDF membranes were blocked with 5% nonfat milk for 30 min at room temperature and then incubated with the primary antibody overnight at 4°C. Subsequently, the membranes were incubated with a horseradish peroxidase-conjugated secondary antibody (1 : 4000 dilution, Thermo Scientific, Waltham, MA, USA) for 1 h at room temperature. Protein expression was detected using an electrochemiluminescence kit (Tanon Science & Technology, Shanghai, China), and images of the blots were obtained by a chemiluminescence imaging system (6100; Tanon Science & Technology). The primary antibodies used are as follows: Collagen 1 (Col1, Abcam); Collagen 3 (Col3, Abcam); and *β*-actin (Bio-Rad).

### 2.8. Histology

Skin tissue from humans and rats was fixed with 4% paraformaldehyde, embedded in paraffin, and sectioned (thickness = 6 *μ*m). Masson staining was conducted by a Masson Stain Kit (Yeasen Biotechnology, Shanghai, China). Picrosirius red staining was performed to visualize collagen fibers using a Picrosirius Red Stain Kit (Yeasen Biotechnology, Shanghai, China). Images were captured using an Olympus BX60 microscope (Olympus, Tokyo, Japan) with a DP25 camera.

### 2.9. Immunofluorescence and Immunohistochemistry Staining

For immunofluorescence (IF), sections were first deparaffinized and then permeabilized and blocked in a solution containing 2% bovine serum albumin, 0.5% Triton X100, and PBS (pH 7.3) for 1 h. Antigen retrieval was conducted according to the antibody datasheet. Cells and sections were incubated with the primary antibody overnight at 4°C and then visualized using a secondary antibody conjugated with fluorophores that absorbed light at 488 nm (1 : 200; Thermo Fisher Scientific, Waltham, MA, USA). Nuclei were stained with DAPI (1 *μ*g/mL; Sigma–Aldrich) for 10 min and mounted in DAKO Mounting Medium (Sigma–Aldrich).

For immunohistochemistry (IHC), sections were preincubated in 3% H_2_O_2_ for 10 min at room temperature (RT). Permeabilization, blocking, and primary antibody incubation were conducted as previously described. Sections were then incubated with a biotinylated secondary antibody for 1 hour at RT. After washing, an avidin-biotin-peroxidase complex was applied (Vector Laboratories, Burlingame, CA, USA) for 1 hour at RT. The sections were washed and incubated with DAB solution for 1 min at RT. Nuclei were stained with hematoxylin.

The primary antibodies used were as follows: CFTR (ab2784, Abcam), Collagen 1 (ab34710, Abcam), and Collagen 3 (ab7778, Abcam). Images were undertaken using a Nikon Ni-U microscope (Nikon, Tokyo, Japan). Image analysis was carried out using ImageJ.

### 2.10. Statistics

All statistical analyses were performed using Prism 6 (GraphPad Software, CA, USA). Statistical differences were determined by a two-tailed Student's *t*-test; *P* values less than 0.05 were considered statistically significant.

## 3. Results

### 3.1. CFTR Was Downregulated in Human Hypertrophic Scars

**Figure 1:**
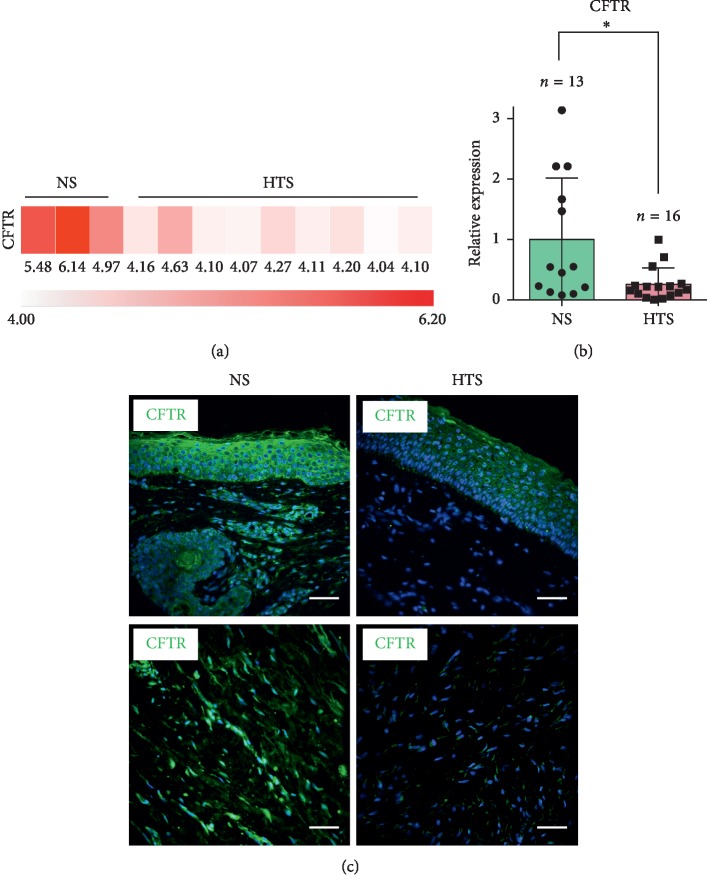
CFTR was downregulated in human hypertrophic scars. (a) CFTR was significantly downregulated in human hypertrophic scars (HTSs) compared to that in human normal skin (NS). Data was generated from microarray data. (b) Real-time RT-PCR data also verified that CFTR was significantly downregulated in human HTS (*P*=0.023). (c) IF staining revealed that CFTR was significantly decreased in human HTS compared to human NS. The upper panels: the epidermis and the superficial layer of the dermis. The bottom panels: the deep layer of the dermis. Scale bar = 50 *μ*m.

First, we examined CFTR gene expression in samples of normal human skin and hypertrophic scars. The expression value of 3 normal skin samples and 9 hypertrophic scar samples generated from microarray data showed a significant downregulation of CFTR in the hypertrophic scar samples (Figure 1(a)). To verify this result, real-time RT-PCR was performed, and a significant downregulation (fold change = 0.256, *P*=0.023, Figure 1(b)) of CFTR was observed in hypertrophic scars (*n* = 16) compared to normal skin (*n* = 13). Immunofluorescence staining for CFTR was then conducted. In accordance with previous results, significantly decreased CFTR protein levels were detected in human hypertrophic scars (HTS) compared to human normal skin (NS) samples (Figure 1(c), top). Moreover, this decrease was observed throughout the dermis in hypertrophic scars (Figure 1(c)).

### 3.2. CFTR Deficiency Can Aggravate the Formation of Hypertrophic Scars and Tissue Inflammatory Responses

**Figure 2:**
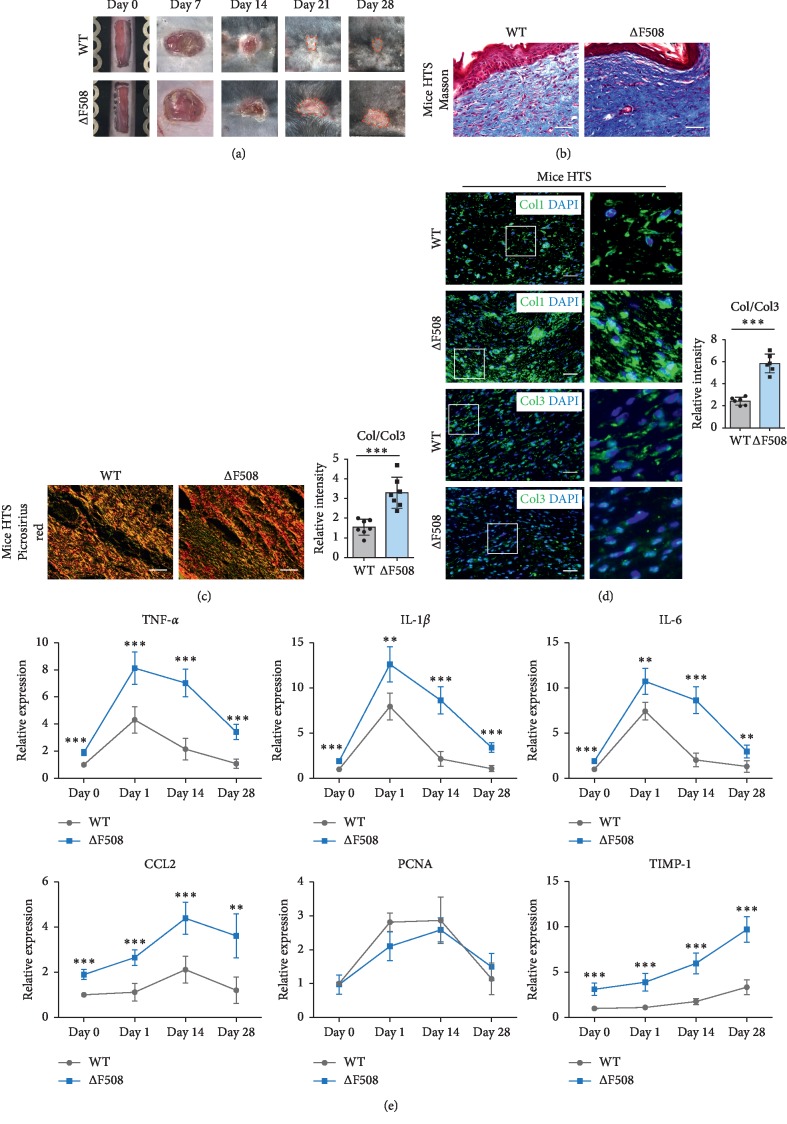
CFTR deficiency can aggravate the formation of hypertrophic scars. (a) Under mechanical loading, wound healing was significantly delayed, and larger hypertrophic scars were detected in ΔF508 mice compared to those in WT mice on day 28. Scar areas were lined out. (b) Masson staining showed that collagen deposition was significantly greater in ΔF508 HTS compared to that in WT HTS. (c) Picrosirius red staining revealed that the ratio of Col1/Col3 was significantly higher in ΔF508 HTS compared to WT HTS. Scale bar = 50 *μ*m. (d): IF staining showed a significantly higher ratio of Col1/Col3 in the HTS of ΔF508 HTS than that of WT HTS. The right panels are the magnification of the left panels. Scale bar = 50 *μ*m. (e) RT-PCR showed that the mRNA expression of TNF-*α*, IL-1*β*, IL-6, CCL-2 was significantly higher in ΔF508 mice during scar formation, whereas the mRNA expression of PCNA was similar. TIMP-1 was significantly higher in ΔF508 mice than that in WT mice, especially in the remodeling phase. ^*∗∗*^*P* < 0.01; ^*∗∗∗*^*P* < 0.001.

To validate whether decreased CFTR expression is involved in the formation of HTSs, we established a hypertrophic scar model in both WT and ΔF508 mice via a mechanical overloading method [8]. After surgery, we found that wound healing was delayed and larger hypertrophic scarring was detected in ΔF508 mice compared to WT mice on day 28 (Figure 2(a)). Masson staining revealed significantly increased collagen deposition in ΔF508 mice compared to WT mice (Figure 2(b)). Sirius red staining showed that the ratio of Col1/Col3 was significantly higher in ΔF508 mice compared to that in WT mice (Figure 2(c)). IF staining showed a 1.40-fold increase of the ratio of Col1/Col3 in the HTS of ΔF508 mice than in WT mice (Figure 2(d)). All these results indicated that CFTR deficiency can aggravate the formation of hypertrophic scars. Furthermore, the results of real-time RT-PCR showed that the mRNA expression of proinflammatory factors (TNF-*α*, IL-1*β*, IL-6, and CCL-2) was significantly higher in ΔF508 mice in all the phases of scar formation, especially in the inflammatory phase, than that in the WT mice. On the contrary, the mRNA expression of PCNA was similar between the two groups (Figure 2(e)). Besides, TIMP-1, an inhibitor of collagen degradation, was significantly higher in ΔF508 mice than in WT mice in all phases, especially in the remodeling phase (Figure 2(e)). These results indicated that ΔF508 mice have undergone more serious inflammatory responses and prolonged inflammatory phase after injury than WT mice, which might be a major course resulting in the delay of wound healing and HTS formation.

### 3.3. CFTR Deficiency in Fibroblasts Can Affect Collagen Production and Deposition under Mechanical Overloading Conditions

**Figure 3:**
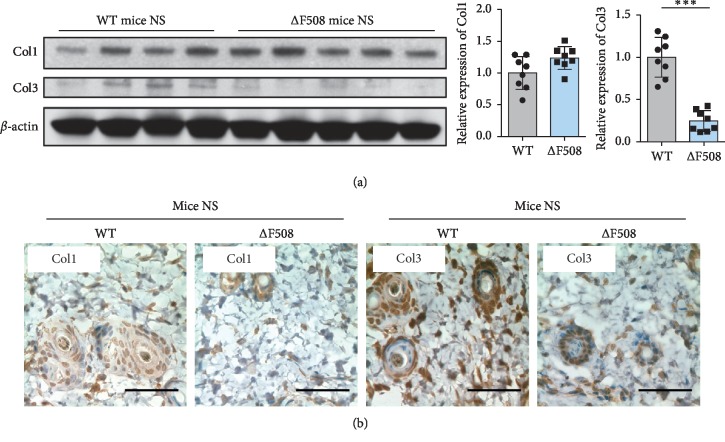
CFTR deficiency can affect collagen production in the normal skin of ΔF508 mice. (a) Western blotting revealed that the expression of Col1 was the same in WT and ΔF508 mice and that the expression of Col3 was significantly lower in ΔF508 mice compared to that in WT mice. ^*∗∗∗*^*P* < 0.001. (b) Consistent with the western blotting results, IHC staining showed no change in Col1 and a significant decrease in Col3 in ΔF508 mice. Scale bar = 50 *μ*m.

To elucidate how CFTR is involved in the formation of HTSs, we first harvested the full-thickness skin tissue of both WT and ΔF508 mice, and the results of western blot (Figure 3(a)) and IHC staining (Figure 3(b)) revealed that there was no difference between the two in the expression of Col1, and the expression of Col3 was significantly lower in ΔF508 mice compared to that in WT mice.

**Figure 4:**
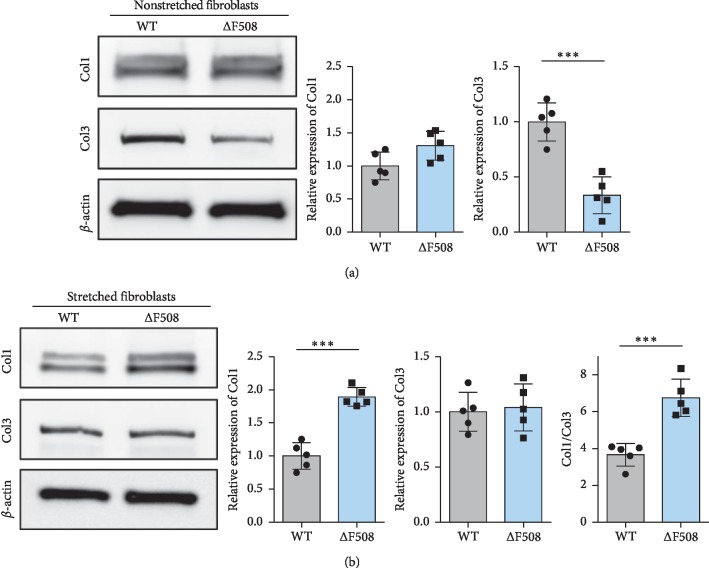
CFTR deficiency in fibroblasts can affect collagen production under mechanical overloading conditions. (a) Without mechanical overloading, the expression of Col1 was the same in WT and ΔF508 fibroblasts, and the expression of Col3 was significantly lower in ΔF508 fibroblasts compared to that in WT fibroblasts. (b) Under mechanical overloading conditions, the expression of Col1 was significantly higher and Col3 was the same, resulting in a higher ratio of Col1/Col3 in ΔF508 fibroblasts compared to that in WT fibroblasts. ^*∗∗∗*^*P* < 0.001.

Then, we dissociated the fibroblasts from both mice and conducted mechanical overloading on the cells. Under nonstretching conditions, the results were the same as those obtained with the whole skin and showed that the expression of Col1 was similar between the two groups and that the expression of Col3 was significantly lower in ΔF508 fibroblasts compared to that of WT fibroblasts (Figure 4(a)). Under mechanical overloading conditions, Col1 of ΔF508 fibroblasts became significantly higher than that of WT fibroblasts, and Col3 levels became the same (Figure 4(b)). These results indicated that the dysfunction of CFTR might contribute to an increase in the ratio of Col1/Col3 and thus promote HTS formation under mechanical overloading.

## 4. Discussion

HTSs are a complex fibroproliferative disorder, and their exact mechanism is still not fully understood. In this study, we first found that CFTR expression was downregulated in human hypertrophic scars at the RNA and protein levels by microarray data analysis, RT-PCR, and immunofluorescence staining. To validate that this downregulation of CFTR was involved in the formation of HTS, we then applied a mechanical overloading intervention on both normal WT mice and CFTR-mutant mice (ΔF508). Mechanical overloading is one of the most common methods used to establish hypertrophic scars in rodents. Previous studies have demonstrated that this method can induce typical HTSs in mice, which are retained for at least 24 weeks [8, 16]. Similarly, our results also showed that HTSs were formed gradually after mechanical overloading in both groups. However, ΔF508 mice showed delayed wound healing and significantly larger HTSs on day 28. Masson staining revealed that there was greater collagen deposition in HTSs, and both Sirius red and IF staining showed a higher ratio of Col1/Col3 in ΔF508 mice. All aforementioned results indicated that the downregulation of CFTR can aggravate the formation of HTSs.

Scar formation typically includes three stages: inflammation, proliferation, and remodeling [17]. During the inflammatory stage, the clotting cascades are activated to form a fibrin clot for hemostasis; meanwhile, the inflammatory cells and macrophages are recruited to the injury area. This stage usually lasts for 3 days. The proliferative stage starts with the formation of granulation tissue, which is made of procollagen, elastin, proteoglycans, and hyaluronic acid and usually lasts for up to 3–21 days. Growth factors secreted from macrophages can induce fibroblast cells to proliferate and differentiate into myofibroblasts, which can lay down collagen. The remodeling stage lasts for 21 days to 1 year, during which the abundant extracellular matrix (ECM) is degraded and Col3 is replaced by Col1 [17, 18]. Among the various types of cells involved in this process, dermal fibroblasts are the key cells regulating HTSs development during the remodeling stage. They can differentiate into myofibroblasts that result in increased ECM synthesis and tissue contraction [19, 20]. Collagen is the major component of the ECM, and its abnormal accumulation leads to scar formation [21]. In the HTS tissues, both Col1 and Col3 were elevated; more importantly, the ratio of Col1/Col3 was significantly increased [22].

In the current study, we found that the CFTR deficiency in fibroblasts could induce lower expression levels of Col3, whereas there was no effect on the expression of Col1, resulting in a higher ratio of Col1/Col3, which indicates increased scar formation. Col3 is a crucial type of collagen in the wound healing process, and it is abundantly produced by fibroblasts and finally replaced by Col1. The dysfunction of CFTR is likely to affect the function of fibroblasts to produce Col3 and impairs re-epithelialization (as reported previously [14]) which leads to the delay of wound healing. However, dysfunction of CFTR alone is not sufficient to directly form hypertrophic scars. Next, we tested the effect of the CFTR deficiency on fibroblasts under mechanical overloading. Interestingly, we found that after mechanical overloading was applied, Col1 of ΔF508 fibroblasts were significantly elevated compared with that of WT fibroblasts, and Col3 remained the same, resulting in a significantly higher ratio of Col1/Col3 (Figure 4). Taken together, our results indicate that the downregulation of CFTR might affect the function of fibroblasts, resulting in a lower content of Col3 and a higher ratio of Col1/Col3. Under mechanical overloading conditions, the fibroblasts are stimulated to produce excessive Col1 and Col3, and a higher ratio of Col1/Col3 induced by dysfunction of CFTR leads to the formation of hypertrophic scars.

On the other hand, we examined the mRNA expression of proinflammatory factors and proliferation markers. We demonstrated that the proinflammatory factors were higher in ΔF508 mice during HTS formation, whereas the proliferation marker was similar. TIMP-1 was significantly higher especially in the remodeling phase. Previously, Huaux et al. reported that proinflammatory and fibrogenic functions of fibroblasts are upregulated in CFTR-mutant mice (ΔF508) [23, 24]. Dong et al. found that ΔF508 mice with defective CFTR exhibited impaired wound healing [14]. Thus, we speculated that CFTR dysfunction leads to elevated inflammation response, impaired wound healing, and inhibited collagen degradation which finally results in HTS formation.

However, the mechanism underlying the effects of CFTR on fibroblast and collagen production was not studied in this study. It is recognized that endoplasmic reticulum (ER) stress can result in the activation of the unfolded protein response and has been implicated in the development of fibrotic diseases in the liver [25], the lung [26], and the kidney [27]. Hsu et al. demonstrated that ER stress could promote fibroblast proliferation and induce pulmonary fibrosis [28]. Heindryckx et al. reported that inhibition of ER stress by an IRE1*α* signaling pathway inhibitor could reduce liver and skin fibrosis in mice [29]. Furthermore, Yang et al. found that the downregulation of CFTR is involved in the activation of ER stress [30]. These aforementioned studies suggest that ER stress might be a crucial process induced by the downregulation of CFTR, contributing to hypertrophic scar formation. The role of ER stress in HTS formation needs to be studied further.

## 5. Conclusions

The current study demonstrated that the downregulation of CFTR might affect the function of fibroblasts, resulting in a lower content of collagen type 3 and a higher ratio of Col1/Col3, and thus aggravate the formation of HTSs in mechanical overloading conditions.

## Figures and Tables

**Table 1:** Primer list.

GAPDH	Forward	5′-TCCCATCACCATCTTCCAG-3′
	Reverse	5′-TCCACCACTGACACGTTG-3′
CFTR	Forward	5′-AAAACTTGGATCCCTATGAAC-3′
	Reverse	5′-GTGGGGAAAGAGCTTCAC-3′
TNF-*α*	Forward	5′-GCCTCTTCTCATTCCTGCTTGT-3′
	Reverse	5′-GGCCATTTGGGAACTTCTCA-3′
IL-1*β*	Forward	5′-GACGGACCCCAAAAGATGAAG-3′
	Reverse	5′-CTCTTGTTGATGTGCTGCTGTG-3′
IL-6	Forward	5′-GAGGATACCACTCCCAACAGACC -3′
	Reverse	5′-CACAACTCTTTTCTCATTTCCACG -3′
CCL-2	Forward	5′-AGCCAGATGCAGTTAACGCC-3′
	Reverse	5′-TTTGGGACACCTGCTGCTG-3′
PCNA	Forward	5′-GGAAGCTTAGAGTAGCTCTCATC-3′
	Reverse	5′-GGGAATTCGTGACAGAAAAGACCTC-3′
TIMP-1	Forward	5′-AACCAGACCACCTTACAGCG-3′
	Reverse	5′-GTCCAATAGTTGTCCGGCGA-3′

## Data Availability

The data used to support the findings of this study are included within the article.
